# B-1b Cells Have Unique Functional Traits Compared to B-1a Cells at Homeostasis and in Aged Hyperlipidemic Mice With Atherosclerosis

**DOI:** 10.3389/fimmu.2022.909475

**Published:** 2022-07-22

**Authors:** Prasad Srikakulapu, Tanyaporn Pattarabanjird, Aditi Upadhye, Sai Vineela Bontha, Victoria Osinski, Melissa A. Marshall, James Garmey, Justine Deroissart, Thomas A. Prohaska, Joseph L. Witztum, Christoph J. Binder, Nichol E. Holodick, Thomas L. Rothstein, Coleen A. McNamara

**Affiliations:** ^1^ Carter Immunology Center, University of Virginia, Charlottesville, VA, United States; ^2^ Cardiovascular Research Center, University of Virginia, Charlottesville, VA, United States; ^3^ Department of Laboratory Medicine, Medical University of Vienna, Vienna, Austria; ^4^ Department of Medicine, University of California San Diego, La Jolla, CA, United States; ^5^ Center for Immunobiology and Department of Investigative Medicine, Western Michigan University Homer Stryker M.D. School of Medicine, Kalamazoo, MI, United States; ^6^ Cardiovascular Division, Department of Medicine, University of Virginia, Charlottesville, VA, United States

**Keywords:** B-1 cells, IgM antibodies, IgHV sequencing, aging, CCR6, atherosclerosis, inflammation

## Abstract

Immunoglobulin M (IgM) to oxidation specific epitopes (OSE) are inversely associated with atherosclerosis in mice and humans. The B-1b subtype of B-1 cells secrete IgM to OSE, and unlike B-1a cells, are capable of long-lasting IgM memory. What attributes make B-1b cells different than B-1a cells is unknown. Our objectives were to determine how B-1b cells produce more IgM compared to B-1a cells at homeostatic condition and to see the differences in the B-1a and B-1b cell distribution and IgM CDR-H3 sequences in mice with advanced atherosclerosis. Here, *in-vivo* studies demonstrated greater migration to spleen, splenic production of IgM and plasma IgM levels in *ApoE^-/-^Rag1^-/-^* mice intraperitoneally injected with equal numbers of B-1b compared to B-1a cells. Bulk RNA seq analysis and flow cytometry of B-1a and B-1b cells identified CCR6 as a chemokine receptor more highly expressed on B-1b cells compared to B-1a. Knockout of CCR6 resulted in reduced B-1b cell migration to the spleen. Moreover, B-1b cell numbers were significantly higher in spleen of aged atherosclerotic *ApoE^-/-^* mice compared to young *ApoE^-/-^* mice. Single cell sequencing results of IgHM in B-1a and B-1b cells from peritoneal cavity and spleen of atherosclerotic aged *ApoE^-/-^* mice revealed significantly more N additions at the V-D and D-J junctions, greater diversity in V region usage and CDR-H3 sequences in B-1b compared to B-1a cells. In summary, B-1b cells demonstrated enhanced CCR6-mediated splenic migration, IgM production, and IgM repertoire diversification compared to B-1a cells. These findings suggest that potential strategies to selectively augment B-1b cell numbers and splenic trafficking could lead to increased and more diverse IgM targeting OSE to limit atherosclerosis.

## Introduction

IgM antibodies that bind to oxidation-specific epitopes (OSE), such as oxidized phospholipids (OxPL) and malondialdehyde (MDA), were discovered over two decades ago. They bind to and block the uptake of oxidized low-density lipoproteins (OxLDL) by macrophages, recognize similar epitopes on apoptotic cells and their levels are increased in hyperlipidemic mice (1–3). Yet translating these findings into effective approaches for inflammatory diseases fueled by OSE, such as nonalcoholic steatohepatitis (4) and atherosclerosis (5), remains to be established. In that regard, a better understanding of the cellular and molecular mechanisms regulating the production of IgM to OSE is needed.

In mice, 80% of the IgM produced comes from B-1 cells (6). In contrast to conventional B-2 cells, B-1 cells are mainly derived from fetal liver. However, Montecino-Rodriguez et al. identified B-1 cell precursors in the bone marrow of adult mice as another source for B-1 cells (7). B-1 cells predominantly reside in serosal cavities, persist throughout life by self-renewal and produce natural IgM with fewer non-template encoded nucleotides in an antigen and T cell-independent manner (8–10). B-1 cells are characterized as CD19^+^B220^lo^CD23^−^CD43^+^IgM^hi^IgD^lo^, B-1 cells are further divided into two subtypes based on cell surface expression of CD5 (CD5^+^ B-1a and CD5^-^ B-1b cells) (11). The prototypical natural antibody, T15, described more than 40 years ago recognizes phosphocholine (PC) antigens on the cell wall of many bacteria (2, 12, 13). Interestingly IgM antibodies found in adipose tissue, atherosclerotic lesions, and plasma from hyperlipidemic mice bear the same classical natural T15 idiotype, anti-PC antibody produced by B-1 cells that provide optimal protection to mice from pneumococcal infection (2, 14–18). As discovery of B-1 cells began with the identification of CD5 (Ly-1) on the surface of cancerous B cells in patients with B cell chronic lymphocytic leukemia (19, 20), the presence of CD5 on murine B cells that were long-lived, self-renewing, producers of polyreactive IgM antibodies has been regarded by many as synonymous with murine B-1 cells. However, it has recently been shown that CD5- B-1 cells (B-1b) produce atheroprotective IgM to OSE in a T cell-independent manner and attenuate diet-induced atherosclerosis in mice (21).

Earlier work on B cell ontology and in infection responses provided evidence that B-1a and B-1b cells are phenotypically, developmentally (9, 11, 22) and functionally distinct (23, 24). Notably, in contrast to B-1a cells, B-1b cells could provide T cell independent long-lasting IgM memory to specific infectious pathogens providing protection from subsequent lethal infection (23–27). Despite these suggestions of a unique and important role for B-1b cells, their phenotype and functional roles in the context of age-related inflammatory diseases such as atherosclerosis are poorly understood.

Here we provide evidence that B-1b cells have greater trafficking to the spleen and IgM production compared to B-1a cells. RNAseq and flow cytometry identify the chemokine receptor CCR6 with significantly higher expression in B-1b compared to B-1a cells and demonstrate distinct Ighv region usage for IgM between these two subtypes. A deeper analysis of VDJ usage of single B-1a and B-1b cells sorted from advanced atherosclerotic aged *ApoE^-/-^* mice revealed significantly more N-region nucleotides and greater diversification of V region usage in B-1b compared to B-1a cells in both peritoneal cavity (PerC) and spleen. Moreover, this diversification/heterogeneity is higher in splenic B-1b cells compared PerC B-1b cells suggesting that the splenic environment is important for the development of antigen specific IgM production from B-1b cells in advanced atherosclerosis.

## Results

### B-1b Cells Have Greater Trafficking to Spleen and IgM Production Compared to B-1a Cells

**Figure 1:**
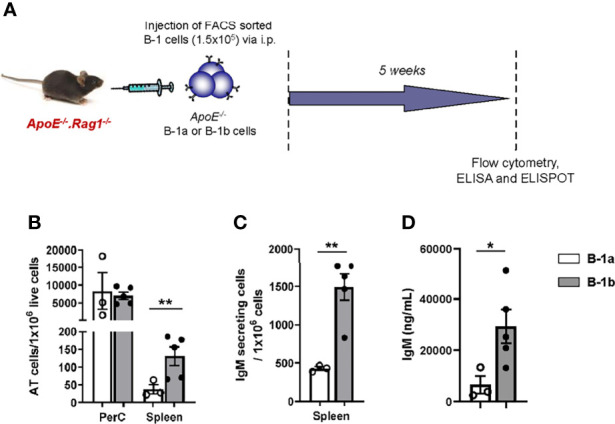
B-1b cells produce more IgM than B-1a cells *in vivo*, likely due to greater accumulation in the spleen. **(A)** Schematic representation of the experiment. FACS sorted B-1a or B-1b cells from *ApoE^-/-^* mice were adoptively transferred (100,000 cells/mouse) into *ApoE^-/-^Rag1^-/-^* mice *via* intraperitoneal injection. **(B)** Adoptively transferred (AT) cells were quantified in peritoneal cavity (PerC) and spleen by flow cytometry and **(C)** IgM secreting cells were quantified in spleen after five weeks of cell transfer. **(D)** Secreted IgM levels in the plasma were measured after five weeks of cell transfer. B-1a transferred mice (n=3) and B-1b transferred mice (n=5). Data from single experiment. Results are represented in mean + SEM, unpaired t-test performed. *p < 0.05, **p < 0.01.

Previous data from our lab demonstrated that B-1b cells produce more IgM than B-1a cells *in vivo* (21). Whether this might be due to differences in survival after injection into the PerC or trafficking to antibody-producing sites is unknown. To address this question, equal numbers (1x10^5^ cells) of FACS-purified PerC B-1a or B-1b cells from *ApoE^-/-^* mice were intraperitoneally (IP) injected into B and T cell deficient *ApoE^-/-^Rag1^-/-^* mice (**Figure 1A**). Flow cytometry analysis of cells in the PerC and spleen of these mice 5 weeks later revealed that the majority of the transferred B cells resided in their homeostatic niche in the PerC. The number of adoptively transferred B cells in the PerC were not different between B-1a or B-1b recipient mice, suggesting equal survival. However, there was a significantly greater number of adoptively transferred B cells in the spleen of B-1b cell recipients compared to B-1a recipients (**Figure 1B**). This data suggests that PerC B-1b cells demonstrate greater trafficking to the spleen compared to B-1a cells. ELISPOT analysis of splenic cells supports the flow data, as more IgM secreting cells accumulated in spleens of mice after B-1b compared to B-1a cell transfer (**Figure 1C**). Moreover, consistent with our previously published data (21), mice that received B-1b cell transfer had significantly higher levels of plasma IgM compared to mice with B-1a cell transfer (**Figure 1D**). To determine if there were differences in the amount of IgM produced by B-1a and B-1b cells from PerC and spleen, equal numbers ofsort-purified B-1a and B-1b cells from 10 weeks old *ApoE^-/-^* mice were cultured for 72 hrs without any exogenous stimulation and total secreted IgM in the culture media was quantified by ELISA. There was minimal IgM measured in the culture media from either B-1 subtype from the PerC. However, B-1b cells spontaneously produced significantly more IgM than B-1a cells when derived from the spleen (**Supplement Figure 1**).

### Chemokine Receptor-6 Regulates B-1b Cell Migration to Spleen

**Figure 2:**
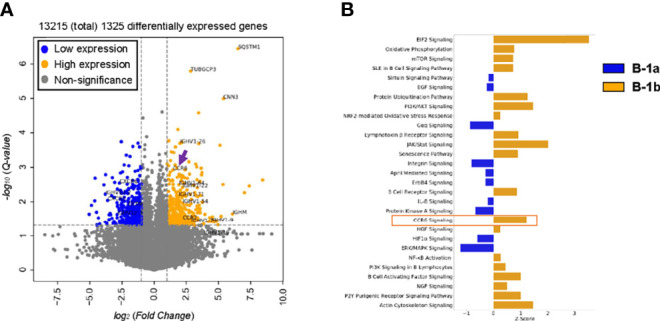
CCR6 expression as well as CCR6 signaling are likely enriched in B-1b cells compared to B-1a cells. **(A)** Volcano plot of differentially expressed genes (FDR < 0.05 and log_2_(FoldChange) > or < 1) enriched in B1a (blue) and B-1b (orange) obtained from bulk RNA sequencing with n = 3 for each group. Arrow indicates CCR6 gene. **(B)** Canonical pathway analysis of differentially expressed genes enriched in B-1a (blue, z- activation score < 0) and B1b (orange, z-activation score > 0) obtained from bulk RNA sequencing.

To determine key differences in gene expression of B-1a and B-1b cells, bulk RNAseq of FAC sort purified PerC B-1a and B-1b cells (PerC gating in **Supplement Figure 1A**) was performed. A total of 13,215 genes were expressed with the majority of them being common to both B-1a and B-1b and 1325 genes (10% of total genes) being differentially expressed (FDR < 0.05, and log_2_(FC) < or > 1) (**Supplementary Table 1**). The volcano plot highlights some of the differentially expressed genes with B1a (blue) and B1b cells (orange) (**Figure 2A** **)**. B-1b cells express unique gene expression signatures compared to B-1a cells with increased expression of *Sqstm1* or *p62*, chemokine receptor-6 (*CCR6*), *CCR7* and immunoglobulin heavy chain (*Ighv*) genes. Notably, canonical pathway analysis showed that, B-1b cells express genes that are involved in CCR6 signaling pathway (**Figure 2B**). Our recent study demonstrated that CCR6 is highly expressed on splenic total B-1 cells compared to total B-1 cells from PerC (28). Since B-1b cells express high levels of CCR6, this CCR6 may regulate the recruitment of B-1b cells to the spleen.

**Figure 3:**
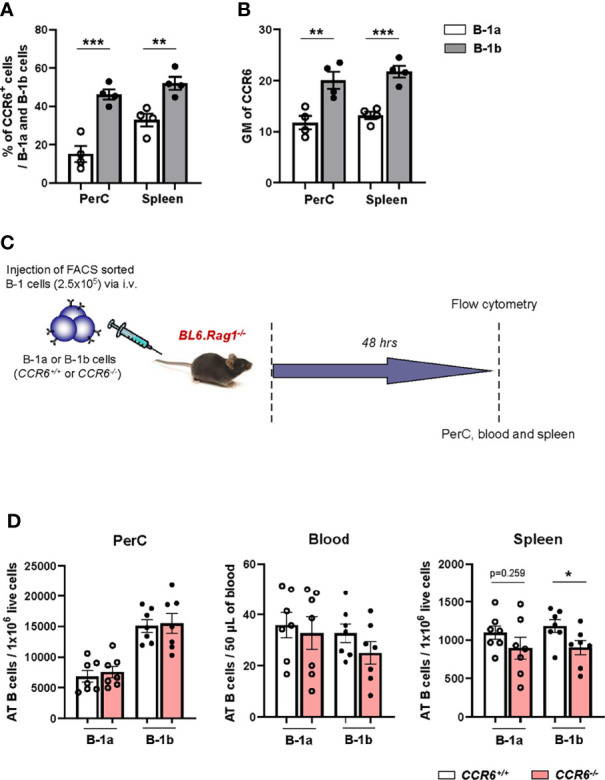
B-1b cells express high amounts of surface expression of CCR6 and this CCR6 is important for B-1b cell migration to spleen. **(A)** Frequency of CCR6^+^ B-1a and B-1b cells in PerC and spleen (n=4 mice). **(B)** quantification of geometric mean (GM) of CCR6 expression on B-1a and B-1b cells in PerC and spleen (n=4 mice). Data is from single experiment. **(C)** Schematic representation of the experiment. FACS sorted B-1a or B-1b cells from *CCR6^+/+^* and *CCR6^-/-^* mice were adoptively transferred (250,000 cells/mouse) into *Rag1^-/-^* mice *via* tail vain injection. **(D)** Adoptively transferred (AT) B cells from live single cells were quantified in peritoneal cavity (PerC), blood and spleen by flow cytometry. Data is from two independent experiments (n=7 mice/group). Results are represented in mean + SEM, unpaired Mann-Whitney test was performed. *p < 0.05, **p < 0.01, ***p < 0.001.

To determine if the CCR6 protein has greater expression in B-1b compared to B-1a cells, we analyzed surface expression of CCR6. Flow cytometry data demonstrated significantly higher percentages of B-1b cells were CCR6^+^ compared to B-1a cells in PerC and spleen (**Figure 3A**). Moreover, the geometric mean of CCR6 expression on B-1b cells was significantly higher compared to B-1a cells in the PerC and spleen (**Figure 3B**). Next, to understand whether CCR6 regulates B-1b cell migration to spleen, we performed adoptive transfer experiments. Two hundred fifty thousand B-1a or B-1b cells were FACS sorted from *CCR6^+/+^* and *CCR6^-/-^* mice and adoptively transferred into B and T cell deficient *Rag1^-/-^* mice *via* tail vein injection. After 48 hrs, CD19^+^IgM^+^ cells from PerC, blood, and spleen were quantified (**Figure 3C**). Presence of the adoptively transferred B cells was observed in the PerC, blood, and spleen of *Rag1^-/-^* mice (**Supplement Figure 2**). There was no difference in CD19^+^IgM^+^ cell numbers in the PerC or blood of mice adoptively transferred with B-1a or B-1b cells from *CCR6^+/+^* and *CCR6^-/-^* mice. However, CCR6 deficiency significantly reduced B cell numbers in the spleen compared to mice that received B-1b cells from *CCR6^+/+^* mice. This effect of CCR6 deficiency was not observed in B-1a cells (**Figure 3D**), providing evidence, that CCR6 regulates B-1b but not B-1a cell recruitment to the spleen. Notably, while the numbers B-1b cells in the spleens after transfer from *CCR6^-/-^* mice was statistically reduced (p=0.037) compared to transfer with *CCR6^+/+^*, there was still evidence for trafficking to the spleen in the absence of CCR6, suggesting that other chemokine receptor may also play a role in B-1b cell splenic trafficking.

### The Spleen Harbors Higher Numbers of B-1b Cells in Aged Mice With Advanced Atherosclerosis Compared to Young Mice

**Figure 4:**
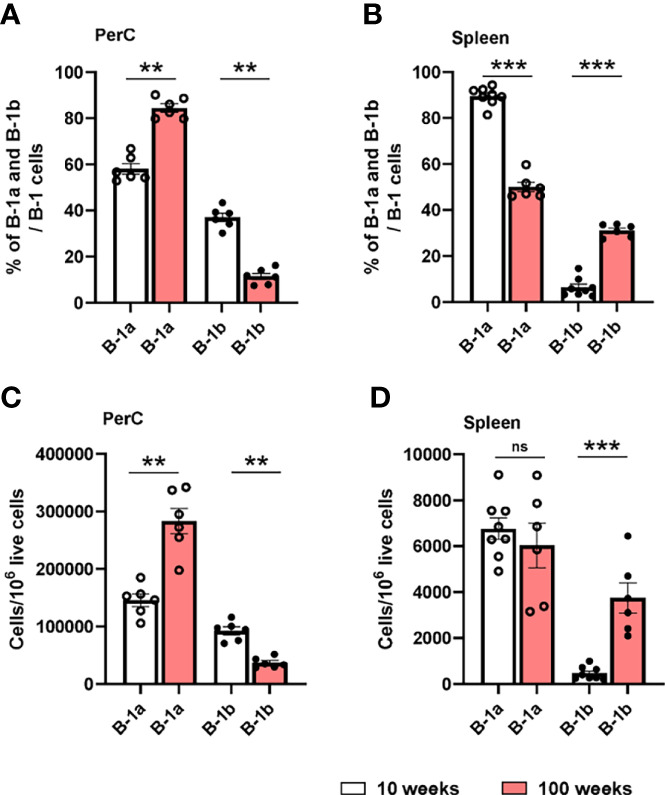
B-1a and B-1b cell distribution in PerC and spleen tissue compartments of both 10- and 100-week-old *ApoE^-/-^* mice. B-1a (CD19^hi^, B220^low^, CD5^+^, IgM^+^) and B-1b (CD19^hi^, B220^low^, CD5^-^, IgM^+^) cell frequencies and numbers were quantified by using flowcytometric analysis in PerC **(A, C)** and spleen **(B, D)** tissue compartments of both 10-week (n=6-8 mice) and 100-week-old (n=6 mice) *ApoE^-/-^* mice. Data from two independent experiments. Results are represented in mean + SEM, unpaired Mann-Whitney test was performed. **p < 0.01, ***p < 0.001.

Next, to understand how age might effect B-1a and B-1b cell distribution in PerC, and spleen, flow cytometric analysis was performed in 10- and 100-week-old, chow diet fed *ApoE^-/-^* mice. 100-week-old, chow diet fed *ApoE^-/-^* mice develop advanced atherosclerosis (**Supplemental Figure 3A**) (29, 30). Flow cytometric data clearly showed that percentages and numbers of B-1a cells were increased and percentages and numbers of B-1b cells were significantly reduced in PerC of 100-week-old mice compared to 10-week-old mice (**Figures 4A, C**). In the spleen, there was significant reduction of B-1a percentages in 100-week-old mice compared to 10-week-old mice (**Figure 4B**). However, there was no difference in B-1a cell numbers between 10- and 100-week-old mice. Intriguingly, B-1b cell percentages and numbers were significantly increased in the spleen of 100-week-old mice compared to 10-week-old mice (**Figures 4B, D**). This data suggests that B-1b cells may be more likely to leave the PerC and migrate to the spleen with aging. We cannot rule out that this age-related change in B-1b cells in the spleen is due to prolonged hyperlipidemia, however we do not see differences in B-1a and B-1b cells percentages in either the PerC or the spleen with hyperlipidemia at homeostasis (**Supplemental Figure 4**).

### B-1b Cells Have Distinct IgHV Repertoires for IgM in Aged Mice With Advanced Atherosclerosis in Both the PerC and Spleen

**Figure 5:**
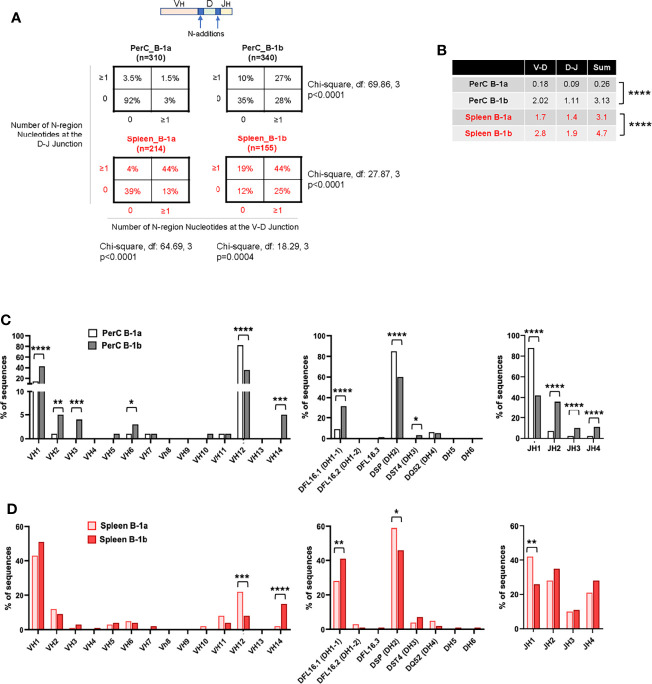
B-1b cells have distinct IgHV repertoires for IgM in aged atherosclerotic *ApoE^-/-^* mice. **(A)** Percentage of total sequences with 0 N-additions at both V-D and D-J junctions (lower left), 1 or more N-additions at both junctions (upper right), or one or more N-addition at either the V-D or D-J junction (upper left or lower right) in the variable region of the immunoglobulin heavy chain of sorted B-1a and B-1b cells from PerC and spleen. **(B)** Average number of N-additions at either the V-D junction, the D-J junction, or the average sum of N-additions at both junctions in sorted PerC and spleen B-1a and B-1b cells. ****P<0.0001 by Mann-Whitney t-test. **(C, D)** Frequency of total sequences utilizing the given V_H_, D, and J_H_ gene segments in the variable region of the immunoglobulin heavy chain from single cell-sorted B-1a and B-1b cells in **(C)** PerC and **(D)** spleen of 100-week-old chow-fed *ApoE^-/-^* mice. *P < 0.05, **P < 0.01, ***P < 0.001, ****P < 0.0001.

Compared to B-1a cells, B-1b cells produce more OSE specific IgM *in vivo* (21). PerC B-1b cells express distinct IgH V repertoire compared to B-1a cells of young C57BL6 mice (31). However, how IgH V repertoire diversity changes between B-1 subsets in PerC and spleen in aged hyperlipemic mice with advanced atherosclerosis is unknown. To determine whether B-1b cells display heterogeneity at the level of the B cell receptor in aged atherosclerotic mice, we sequenced the IgM heavy chain variable region (IgMV_H_) of sorted single B-1a and B-1b cells from the PerC and spleen of 100-week-old, chow-fed *ApoE^-/-^* mice (**Supplement Figure 3**). First, we compared PerC B-1b IgM repertoire data with our recently published PerC B-1a data (32) from the same mice. Results provide the first evidence of B-1a and B-1b heterogeneity in the IgM repertoire in PerC in aged atherosclerotic mice. PerC B-1b cells displayed significantly more N-additions, with 27% of sequences containing ≥1 N-addition at both junctions and low frequency of sequences (35%) containing 0 N-addition at both junctions compared to 1.5% and 92% of sequences respectively in PerC B-1a cells (P<0.0001 by 2x4 χ^2^ analysis) (**Figure 5A**). Intriguingly, splenic B-1b cells contained even more N-additions than PerC B-1b, with 44% of sequences containing ≥1 N-addition at both junctions (P<0.001 by 2x4 χ^2^ analysis compared to PerC B-1b (27%); **Figure 5A**). Splenic B-1a cells also displayed frequent N-additions, with 44% of sequences containing ≥1 N-addition at both junctions, yet a large fraction (39%) of splenic B-1a sequences also contained 0 N-additions at both junctions (P<0.0001 by 2x4 χ^2^ analysis compared to Spleen B-1b (12%); **Figure 5A**). Furthermore, quantifying the number of N-additions in PerC B-1a sequences indicated an average sum of 0.26 N-additions at the V-D and D-J junctions in PerC B-1a cells (32) (**Figure 5B**), in accordance with the finding that many PerC B-1a sequences lacked N-additions completely. In contrast, PerC B-1b, splenic B-1b, and splenic B-1a sequences contained significantly more N-additions compared to PerC B-1a cells, with splenic B-1b cells containing the highest number of N-additions at both the V-D junction, D-J junction, and sum of both junctions (**Figure 5B**). These findings indicate a shift away from germline, with junctional heterogeneity increasing from PerC B-1a < PerC B-1b < splenic B-1a < splenic B-1b.

**Figure 6:**
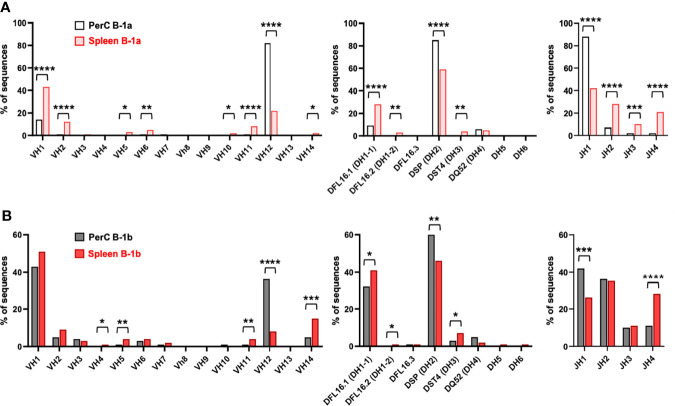
B-1a and B-1b cell IgM CDR-H3 sequencing in the 100-week-old mice. Frequency of total sequences utilizing the given V_H_, D, and J_H_ gene segments in the variable region of the immunoglobulin heavy chain from single cell-sorted **(A)** B-1a cells and **(B)** B-1b cells in both PerC and spleen of 100-week-old chow-fed *ApoE^-/-^* mice. *P < 0.05, **P < 0.01, ***P < 0.001, ****P < 0.0001.

Analysis of VDJ gene segment usage also indicated repertoire differences between B-1a and B-1b in both PerC and spleen. In contrast to PerC B-1a cells, in which >80% of sequences utilized V_H_12, D_H_2, and J_H_1 family gene segments (32), PerC B-1b sequences utilized V_H_1, V_H_2, V_H_3, V_H_6, V_H_14, D_H_1-1, D_H_3, J_H_2, J_H_3 and J_H_4. (**Figure 5C**). Intriguingly, not many differences were observed between Splenic B-1a and B-1b cells. Comparison of splenic B-1a VDJ gene usage to splenic B-1b dataset revealed that splenic B-1a cells also predominantly utilized V_H_1 family genes, but had increased usage of V_H_12, D_H_2, and J_H_1 family genes, while splenic B-1b cells displayed increased usage of V_H_14 and D_H_1-1 family genes (**Figure 5D**). Representation of these data comparing B-1 subtypes in PerC vs spleen (**Figures 6A, B**) highlights niche-specific differences. In contrast to PerC B-1a VDJ usage (V_H_12, D_H_2, and J_H_1), most of the splenic B-1a sequences used V_H_1 family genes (40%). In addition, splenic B-1a cells expressed greater usage of other VDJ family genes: V_H_2, V_H_5, V_H_6, V_H_10, V_H_11, V_H_14, D_H_1-1, D_H_1-2, D_H_3, J_H_2, J_H_3 and J_H_4 (**Figure 6A**). A majority of splenic B-1b cells expressed V_H_1 family genes, and also had significantly greater usage of V_H_4, V_H_5, V_H_11 and V_H_14 genes compared to PerC B-1b sequences. PerC B-1b sequences preferentially utilized D_H_2 genes, while splenic B-1b cells had fairly equivalent usage of D_H_1-1 and D_H_2 genes. Analysis of J segment usage revealed increased expression of J_H_1 segments by PerC B-1b cells and increased usage of J_H_4 segments by splenic B-1b cells (**Figure 6B**). Importantly, analysis of CDR-H3 amino acid sequences revealed a high frequency of replicate sequences present in more than one B-1a or B-1b cell in these compartments. In contrast to PerC B-1a cells, in which 92% of sequences were replicates (32), 65% of PerC B-1b, 56% of splenic B-1a, and 39% of splenic B-1b cells expressed replicate sequences, again demonstrating that heterogeneity increases in the order: PerC B-1a < PerC B-1b < splenic B-1a < splenic B-1b. All replicate CDR-H3 sequences are given in **Supplementary Table 2**.

## Discussion

A large body of evidence supports the role of OxLDL as a mediator of the inflammatory responses that propagate atherosclerotic lesion formation (33–36). OSEs are dominant targets of B-1 cell derived innate IgM antibodies in both humans and mice (16, 37, 38), and recent elegant work using E06-scFv transgenic mice targeting OxPL clearly demonstrates the potent anti-inflammatory effect of blocking the actions of OxPL in limiting atherosclerosis (5). Though B-1a and B-1b subsets are both atheroprotective through the production of IgM to OSE (21, 39), they are developmentally and functionally distinct (10, 11, 22–24). Our previous studies demonstrated preferential accumulation of B-1b cells in atherosclerotic aorta during aging. Aorta tertiary lymphoid organs adjacent to atherosclerotic plaques in aged *ApoE^-/-^* mice harbor significantly higher numbers of B-1b (> 80% of B-1 cells) compared to B-1a cells (30). These B-1b cells produce atheroprotective IgM and anti-inflammatory cytokines IL-10 and TGFβ, providing a potential local mechanism of regulating artery inflammation (30).

B-1a cells produce greater amounts of total IgM and IgM to OSE compared to B-1b cells when stimulated with LPS *in vitro* (21). This may be due to differentiation of B-1a cells into CD138^+^ plasmablasts or plasma cells after TLR stimulation (40) (unpublished data) or due to triggering of TLR, resulting in IgM-BCR reorganization and differentiation into IgM secreting plasmablasts (41). Yet B-1b cells produce greater amounts of total IgM and IgM to OSEs than B-1a cells *in-vivo* (21), providing evidence that the biology of B-1b cells is different than B-1a cells. In the present study, we sought to determine if this *in vivo*-specific effect may be due to unique properties of B-1b versus B-1a cells. Indeed, we demonstrated recruitment of higher numbers of B-1b compared to B-1a cells to the spleen 5 weeks after adoptive transfer of equal numbers of cells. Moreover, IgM secreting cell numbers were higher in the spleen. This may be due to differentiation of adoptively transferred B-1a or B-1b cells into IgM secreting plasmablasts or plasma cells in the spleen. In addition, cell culture data showed that spleen-derived B-1b cells secrete more total IgM compared to B-1a cells *ex vivo*, providing evidence that B-1b cells have greater IgM production on a per cell basis compared to B-1a cells in the splenic niche. As the spleen is one of the major IgM antibody producing sites, higher levels of plasma total IgM and IgM to MDA-LDL in *ApoE.Rag1^-/-^* mice after 4 weeks of adoptive transfer of B-1b cells compared to mice that received equal numbers of B-1a cells (21) may reflect a combination of both increased trafficking and increased production once in the spleen.

Chemokine/chemokine receptors interactions is a major mechanism regulating trafficking of immune cells throughout the body and CCR6 has been shown to regulate B-1 cell mediated atheroprotection in mice (28). In this present study, we found that B-1b cells express significantly more CCR6 than B-1a cells and that CCR6 regulates B-1b cell recruitment to the spleen. In addition, RNA-seq data shows upregulation of CCR7 in B-1b cells, suggesting that other chemokine receptor may regulate B-1b cell migration to the spleen. However, further studies are needed to carefully identify other needed receptors and confirm their function in regulating splenic trafficking of B-1b cells. Interestingly, we previously, demonstrated that CX-Chemokine Receptor-4 (CXCR4) expression on B-1a but not B-1b cells was responsible for migration and IgM secretion to the bone marrow (32), suggesting that different chemokine receptors may be involved in regulation of recruitment or migration of specific B-1 subsets to distinct tissue compartments.

In addition to chemokine receptors, we observed other highly expressed genes in B-1b cells. Interestingly, *Sqstm1* or *p62*, a scaffold and ubiquitin-binding protein, was found to be the most highly enriched gene. Recently, using loss and gain of function studies of *p62*, we have demonstrated that *p62* in B-1b cells positively regulates B-1b cell proliferation, IgM production and atheroprotection (42). Canonical pathway analysis showed that in addition to CCR6 signaling pathway, B cell activation factor (BAFF) signaling, B cell receptor (BCR) mediated signaling and eukaryotic initiation factor 2 (EIF2) signaling pathways were upregulated in B-1b cells. Previous studies demonstrated that BAFF-BAFF receptor signaling is important for B cell maturation and development in periphery (43). In our recent study we showed that, B-1b cells express high levels of BAFF receptor on cell surface compared to B-1a cells and also, stimulation with BAFF increased proliferation in B-1b cells but not in B-1a cells (42), suggesting that B-1b cells are highly proliferative in response to BAFF.

Mielke N, et al. demonstrated that EIF2 signaling is important for B cell development, BCR mediated proliferation and Ig secretion (44). Moreover, it is well described that BCR signaling is essential for conventional B cell survival and development, and antibody production in both physiological and pathological conditions (45). In our canonical pathway analysis, we observed upregulation of both EIF2 signaling and BCR signaling pathways in B-1b cells. This data suggests that upregulation of EIF2 and BCR signaling pathways in B-1b cells may be important for B-1b cell survival, proliferation, and IgM secretion. Further studies are needed to understand these pathways mechanism. Intriguingly, while the above-mentioned signaling pathways were reported to be important in conventional B cell development (44, 45), our RNA seq data showed upregulation of these pathways in B-1b cells, suggesting that B-1b cells have features of both innate B-1 and adaptive B-2 cells.

In addition to enhanced trafficking to the spleen and increased total IgM production, B-1b cells show more diversity than B-1a cells in the context of the IgM repertoire. The % of 0 N-additions at both V-D and D-J junctions is significantly lower in B-1b cells compared to B-1a cells in both PerC and spleen. Differences were also observed in VDJ gene usage between B-1a and B-1b in PerC and spleen. Although, it is interesting to note that the expected high percentage of 0 N-additions, VH12 usage and common CDR H3 that characterize natural antibody-producing B-1a cells is significantly more prominent in B-1a cells in PerC compared to the spleen. Whether this is due to differential origin or developmental niche of these B-1a cells, or the effect of niche-specific selection is unknown. That adoptive transfer into the PerC of PerC-derived B-1a cells results in trafficking of these cells to the spleen at least in part supports the potential for niche-specific selection or antigen-specific sculpting of their repertoire in the spleen.

Notably, both B-1a and B-1b cells in the spleen undergo high intrinsic selection pressures. PerC B-1 cells display high usage of V_H_11 after LPS stimulation (46) and both VH11 and VH12 have anti-phosphatidylcholine specificity (31, 47). These anti-phosphatidylcholine IgM have the capacity to clear microbial infection in mice (48). Previous data in young female C57BL6 mice have demonstrated that PerC B-1a cells highly use VH11 (60%) and low frequency use of VH12 (10%) (31). However, in this current study PerC B-1a specifically used VH12 (80%). These data are consistent with previously published data showing selective increase in VH12 but not VH11 usage in PerC B-1 cells during aging (49). Moreover, consistent with previous reports in young C57BL/6 mice (31), PerC B-1b and splenic B-1a cells highly use VH1, which is involved in the development of anti- phosphocholine specific antibodies (50–52) and is highly used by both PerC and splenic conventional B cells (47). These data suggest that B-1a cells, particularly those utilizing VH12 and VH1 in PerC and spleen respectively, were selected by both aging and in response to atherosclerosis progression in *ApoE^-/-^* mice. Interestingly, PerC B-1b equally utilize VH1 (40%) and VH12 (40%), while splenic B-1b cells highly utilize VH1 (50%) and VH14 (15%), demonstrating that unlike B-1a cells, B-1b cells in both compartments predominantly utilize VH1. These data suggest that B-1b cells either primarily utilize VH1 or it is a selective phenomenon occurring during aging and disease progression.

Our recently published data showed that PerC B-1a cells have 92% of 0 N-additions and 70% of CDR-H3 sequences accounted for by a single amino acid sequence (AGDYDGYWYFDV) (32), which is the 2^nd^ most frequent amino acid sequence in splenic B-1a cells (9.3%). Moreover, that splenic B-1a cells are fairly evenly split between sequences containing >1 N-addition at both junctions (44%), and sequences containing 0 N-additions at both junctions (39%), fits with what has been previously shown by the Herzenberg group, that the splenic B-1a population consists of both a resident B-1a population, as well as a population of B-1a cells that have migrated from the PerC (40). The sequences containing 0 N-additions may be B-1a cells migrating from the PerC. Another possibility is migration of B-1a cells from bone marrow origin, where B-1a cells display 46% and 32% of 0 and >1 N-additions respectively at both junctions (32). Moreover, the top CDR-H3 sequence (AREVTTMYYFDY) in splenic B-1a cells (14%) is the 2^nd^ top sequence in BM B-1a cells. With one amino acid change in the top PerC B-1a sequence, 26.8% of PerC B-1b cells display this sequence (AGD**R**DGYWYFDV) as the top sequence. Intriguingly, this PerC B-1b sequence is not highly (1.3%) accounted for in splenic B-1b cells. However, the 2^nd^ top (15.3%) amino acid sequence (AREDYYGSSYYFDY) in PerC B-1b, is expressed as the top (9.7%) sequence by splenic B-1b cells, which is expressed in very low frequency (0.9%) in splenic B-1a and not expressed in PerC B-1a cells. These data suggest that similar to PerC B-1a cells, PerC B-1b cells may also migrate to spleen in response to specific signals in aged *ApoE^-/-^* mice. Indeed, our adoptive transfer studies demonstrate that PerC B-1a and B-1b cells injected into the PerC of *Rag1^-/-^* mice are found in the spleen strongly supports this notion.

In summary, similar to conventional B-2 cells, B-1b cells use more VH1 genes, and more N-additions in V-D and D-J junctions, suggesting that B-1b cells may bridge between innate B-1a cells and adaptive B-2 cells.

## Materials and Methods

### Animals

All animal protocols were approved by the Animal Care and Use Committee at the University of Virginia. C57BL/6J, CC-chemokine receptor-6 deficient (*CCR6^-/-^*) and Lymphocyte deficient (*Rag1^-/-^*), and Apolipoprotein E deficient mice (*ApoE* ^-/-^) mice were purchased from Jackson Laboratory. *Rag1* ^-/-^ mice were bred with *ApoE^-/-^* to generate *ApoE^-/-^Rag1* ^-/-^ mice. All purchased mice were on a pure C57BL/6J background and those bred were backcrossed 10 generations to C57BL/6J mice. All mice were given water *ad libitum* and either a standard chow diet (Tekland, 7012) or western diet (WD) (Tekland, 88137). Mice were euthanized in all experiments with CO_2_ inhalation. Only male mice were used for all experiments.

### Preparation of Tissues for Flowcytometry and Cell Sorting

Peritoneal cells and spleen cells were harvested and brought into single cell suspension as previously described (21). Cells were blocked for Fc receptors then stained for cell surface markers using fluorescently conjugated antibodies for 20 minutes at 4°C. For flow cytometry, cells were washed in PBS and stained with a fixable live/dead stain diluted in PBS for 20 minutes at 20°C then fixed in 2% PFA in PBS for 10 minutes prior to re-suspending in FACS buffer. For FAC sorting, cells were re-suspended in DAPI live/dead stain before sorting. Flow cytometry antibodies: CD5 (53-7.3), CD19 (1D3), CD23 (B3B4), CD43 (S7), B220/CD45R (RA3-6B2), IgD (11-26.2a), IgM (II/41, R6-60.2), and CCR6 (29-2L17) were purchased from eBioscience, BD Bioscience, and Biolegend. Live/Dead discrimination was determined by LIVE/DEAD fixable yellow or Aqua staining (Invitrogen) or DAPI. Cells were run on a CyAN ADP (Beckman Coulter) or Attune NXT (Invitrogen) or sorted on an Influx cell sorter (BD Bioscience) and analyzed with FlowJo software (Tree Star inc). All gates were determined using fluorescence minus one (FMO) control.

### Bulk RNA Sequence Analysis

FAC sorted peritoneal B-1a and B-1b cells from C57BL/6 mice were used for RNA extraction by using Qiagen RNeasy Plus kit. The purified RNAs were stored at -80°C and sent to a Novogene to perform sequencing. RNA sequences in raw FASTQ data files were obtained from Novogene. Sequencing reads were aligned to reference genome (GRCm38/mm10) using HISAT2. The annotated sequences were then quantified and assembled by using StringTie, and differentially expressed genes were analyzed using R Ballgown package. Volcano plots of differentially expressed genes were visualized by using the python bioinfokit package. Ingenuity Pathway Analysis was performed on all the annotated RNA to analyze for differentially regulated canonical pathways.

### Single Cell IGVH Sequencing for IgM

Single cell sorting of PerC and splenic B-1 subsets were performed and IGVH sequencing for IgM was performed as we previously described (32).

### Adoptive Transfer of B-1a and B-1b Cells Into Rag1^-/-^ Mice

Following FAC sorting, as described above, 1x10^5^ B-1a or B-1b cells from young *ApoE* ^-/-^ mice were transferred intra-peritoneal into 8-week-old *ApoE* ^-/-^ *Rag1* ^-/-^ hosts. Mice were maintained on normal chow diet for 5 weeks. Four weeks after the cell transfer, mice were euthanized, flow cytometry and ELISPOT experiments were performed in PerC and spleen. Plasma IgM levels were analyzed by ELISA.

For short-term adoptive transfer experiment, following FAC sorting, 2.5x10^5^ B-1a or B-1b cells from 8-week-old CCR6^+/+^ and CCR6^-/-^ mice were transferred into 8-week-old *Rag1* ^-/-^ hosts *via* tail vein injection. After 48 hours, the mice were euthanized, and flow cytometry was performed to quantify adoptively transferred B-1a and B-1b cells in PerC, blood, and spleen.

### Enzyme-Linked Immuno Spot Assay

Total IgM secreting B cells were determined by ELISPOT method as we previously described (21, 30).

### Enzyme-Linked Immunosorbent Assay

Total IgM antibodies in mouse plasma were determined by ELISA as we previously described (21).

### Statistics

Unpaired t-test was used for analyzing data with normal distribution and equal variance. For data sets with non-normal distribution, Mann-Whitney test was used. In RNAseq analysis, false discovery rate (FDR) method was used to perform p-value corrections for DEG analysis. Results are displayed containing all replicated experiments, and values shown are mean ± SEM. Data were analyzed using Prism 8 (GraphPad Software, Inc).

## Data Availability Statement

The dataset presented in the study are deposited in the GEO repository, accession number GSE207011.

## Ethics Statement

The animal study was reviewed and approved by UVa Animal Care and Use Committee (ACUC) protocol.

## Author Contributions

PS designed and performed murine experiments, analyzed and interpreted data, and wrote the manuscript. TP performed RNAseq analysis. AU, VO and MM performed murine experiments. JG performed RNA isolation for RNAseq experiments. SB, JD, TAP, JW, CB provided intellectual input. NH and TR involved in IgH V sequencing, analysis and provided intellectual input. CM conceived the project, designed experiments, supervised the study, interpreted the data, and edited the manuscript.

## Funding

This work was supported by 1R01 HL107490, 1R01 HL136098, Project 3 of P01 HL055798 and P01 HL136275-01 (CM). PS was supported by AHA Career Development Award 18CDA34110392. Research reported in this publication was supported by the National Institute of Allergy and Infectious Diseases of the National Institutes of Health under Award Number R01AI154539 (NH). The content is solely the responsibility of the authors and does not necessarily represent the official views of the National Institutes of Health.

## Conflict of Interest

JW is co-inventor and receive royalties from patents owned by UCSD on oxidation-specific antibodies and of biomarkers related to oxidized lipoproteins, and JW is a co-founder of Oxitope, Inc and Kleanthi Diagnostic. JW is a consultant to Ionis Pharmaceuticals.

The remaining authors declare that the research was conducted in the absence of any commercial or financial relationships that could be construed as a potential conflict of interest.

## Publisher’s Note

All claims expressed in this article are solely those of the authors and do not necessarily represent those of their affiliated organizations, or those of the publisher, the editors and the reviewers. Any product that may be evaluated in this article, or claim that may be made by its manufacturer, is not guaranteed or endorsed by the publisher.

## Supplementary Material

Supplementary Figure 1 Splenic B-1b cells produce more IgM in *ex-vivo* **. (A)** B-1a and B-1b cells from PerC and spleen were FAC sorted from 10-week-old *ApoE^-/-^* mice (n=4 mice), cultured for 72 hrs without any exogenous stimulation. **(B)** secreted IgM levels in culture media were quantified by ELISA. Results are represented in mean ± SEM, unpaired student t-test. *p<0.05, ns: not significant.

Supplementary Figure 2 Flow cytometric gating strategy for adoptively transferred B cells. After 48 hrs of adoptive transfer of B-1a or B-1b cells, adoptively transferred B cells (AT B cells: CD19^+^IgM^+^) were gated from total live CD45^+^ cells in PerC, blood and spleen **(A–C)**. PBS injected mice used as control group for AT B cell gating. B-1a and B-1b cells were gated from CD19^+^IgM^+^ B cells for the confirmation.

Supplementary Figure 3 Schematic of experimental flow for B-1a and B-1b single cell IgM CDR-H3 sequencing in the 100-week-old mice. **(A)** B-1a and B-1b cells from spleen and peritoneal cavity of five 100-week-old chow-fed *ApoE^-/-^* mice were single-cell sorted into 96 well plates containing lysis buffer. cDNA was prepared and then PCR was done for Ig heavy chain variable part of IgM. PCR products were sequenced (Genewiz) and analyzed using an online sequence analysis tool, IMGT/HighV-Quest, and only sequences with >90% homology to VDJ regions were included. **(B)** Flow cytometric gating strategy for B-1a and B-1b single cell sorting in PerC and spleen tissue compartments of 100-week-old *ApoE^-/-^* mice.

Supplementary Figure 4 Hyperlipidemia did not alter the frequencies of B-1a and B-1b cells at homeostasis. Flowcytometric analysis was performed in C57BL6 wild type and *ApoE^-/-^* mice at age of 10–12-week-old mice (n=5 mice/genotype) to understand the hyperlipidemic effect on B-1a and B-1b cells numbers in PerC and spleen. Results are represented in mean ± SEM, unpaired Mann-Whitney test was performed.

Supplementary Table 1 List of differentially expressed genes in B-1b over B-1a cells from bulk RNAseq analysis.

Supplementary Table 2 Replicate CDR-H3 amino acid sequences (those present more than once) and frequency present in sorted PerC B-1a, B-1b, splenic B-1a and B-1b. Asterisk (*) in PerC B-1a CDR-H3 sequence indicates stop codon.
