# Supplementary figures and images for: B-1b Cells Have Unique Functional Traits Compared to B-1a Cells at Homeostasis and in Aged Hyperlipidemic Mice With Atherosclerosis
Source: Front Immunol. 2022 Jul 22;13:909475. doi: 10.3389/fimmu.2022.909475 (PMC9353528; doi:10.3389/fimmu.2022.909475)

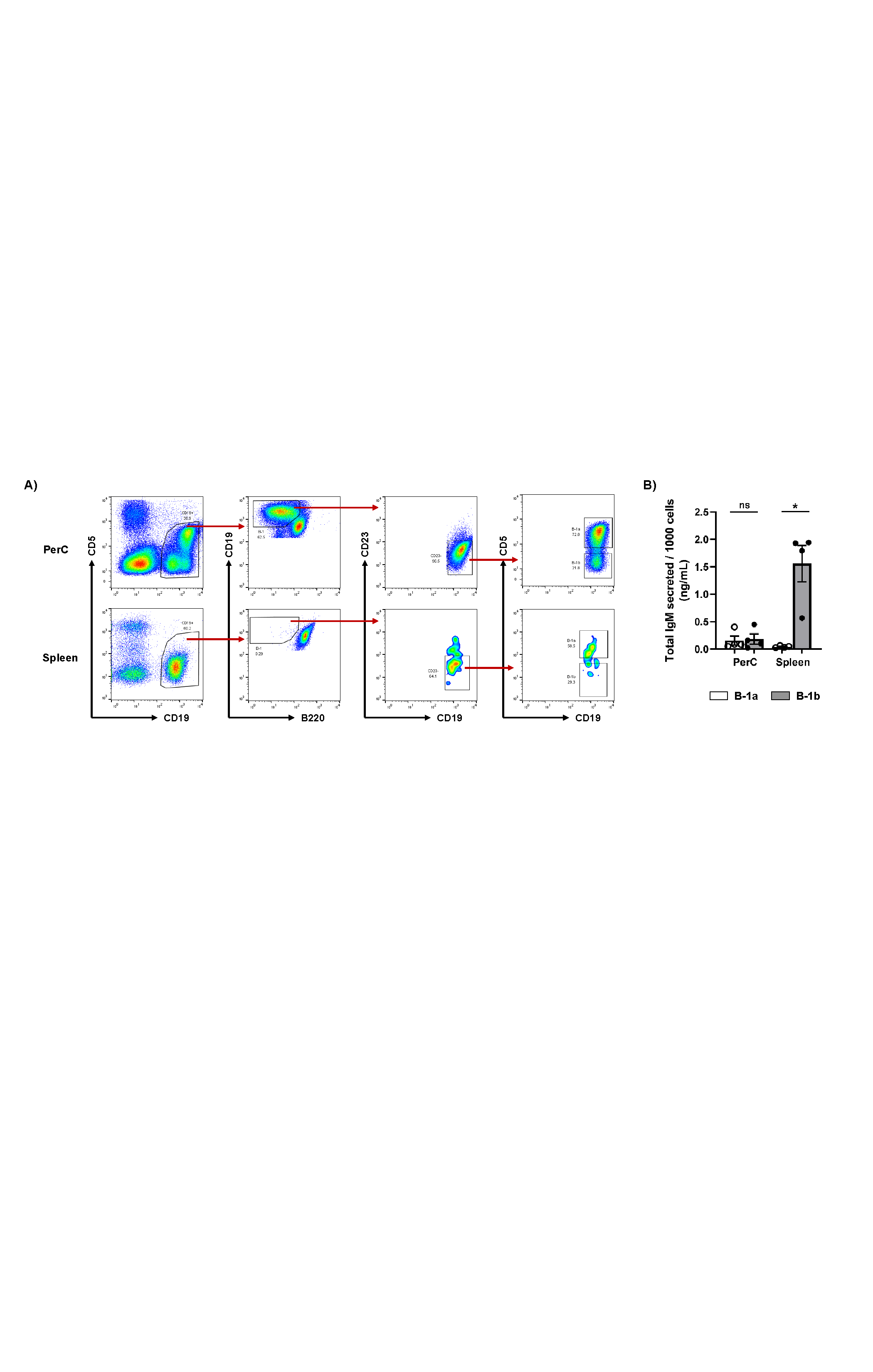

Supplement: Supplementary Figure 1 — Splenic B-1b cells produce more IgM in ex-vivo . (A) B-1a and B-1b cells from PerC and spleen were FAC sorted from 10-week-old ApoE-/- mice (n=4 mice), cultured for 72 hrs without any exogenous stimulation. (B) secreted IgM levels in culture media were quantified by ELISA. Results are represented in mean ± SEM, unpaired student t-test. *p<0.05, ns: not significant. [file Image_1.tif]

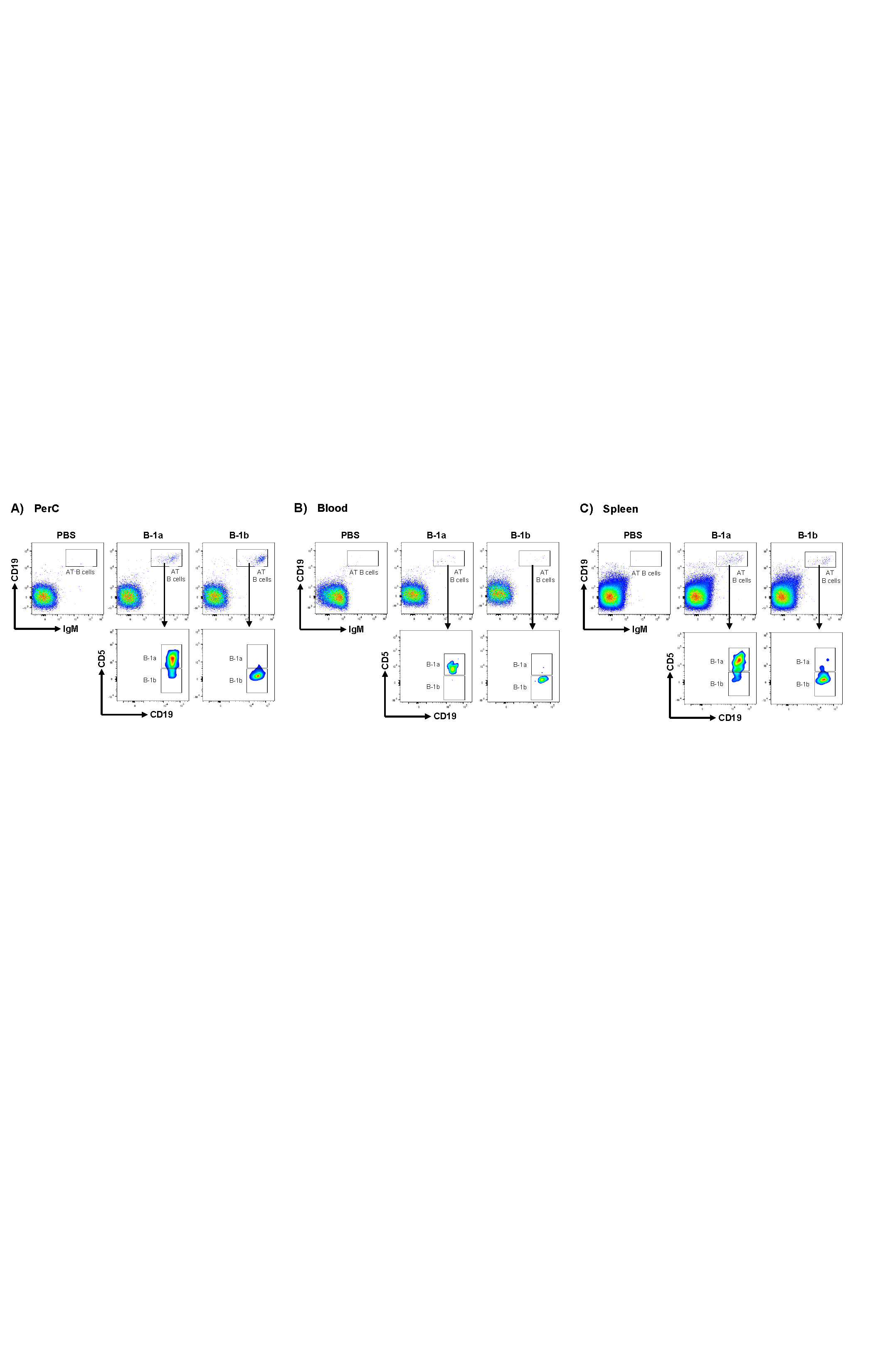

Supplement: Supplementary Figure 2 — Flow cytometric gating strategy for adoptively transferred B cells. After 48 hrs of adoptive transfer of B-1a or B-1b cells, adoptively transferred B cells (AT B cells: CD19+IgM+) were gated from total live CD45+ cells in PerC, blood and spleen (A–C). PBS injected mice used as control group for AT B cell gating. B-1a and B-1b cells were gated from CD19+IgM+ B cells for the confirmation. [file Image_2.tif]

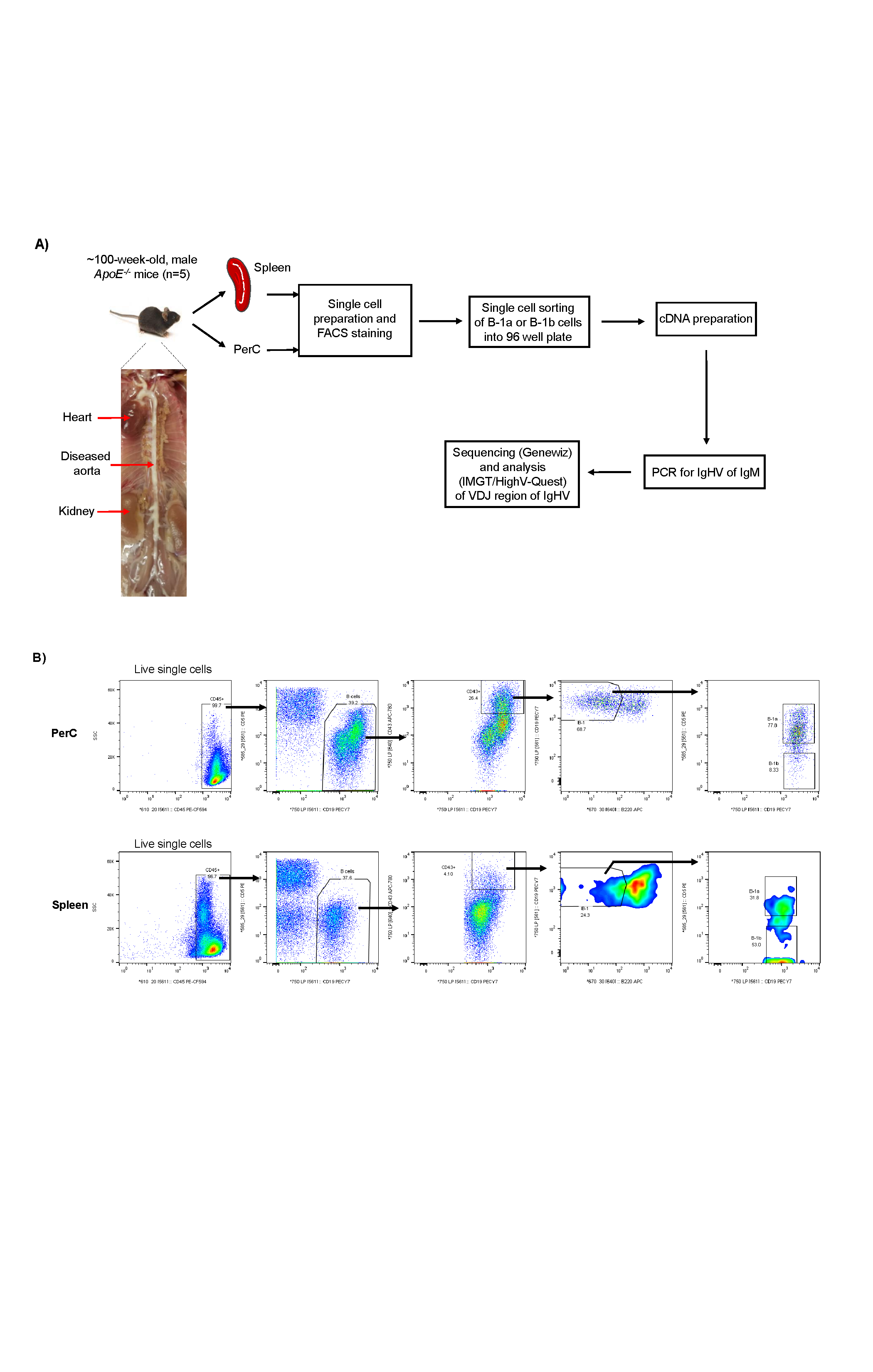

Supplement: Supplementary Figure 3 — Schematic of experimental flow for B-1a and B-1b single cell IgM CDR-H3 sequencing in the 100-week-old mice. (A) B-1a and B-1b cells from spleen and peritoneal cavity of five 100-week-old chow-fed ApoE-/- mice were single-cell sorted into 96 well plates containing lysis buffer. cDNA was prepared and then PCR was done for Ig heavy chain variable part of IgM. PCR products were sequenced (Genewiz) and analyzed using an online sequence analysis tool, IMGT/HighV-Quest, and only sequences with >90% homology to VDJ regions were included. (B) Flow cytometric gating strategy for B-1a and B-1b single cell sorting in PerC and spleen tissue compartments of 100-week-old ApoE-/- mice. [file Image_3.tif]

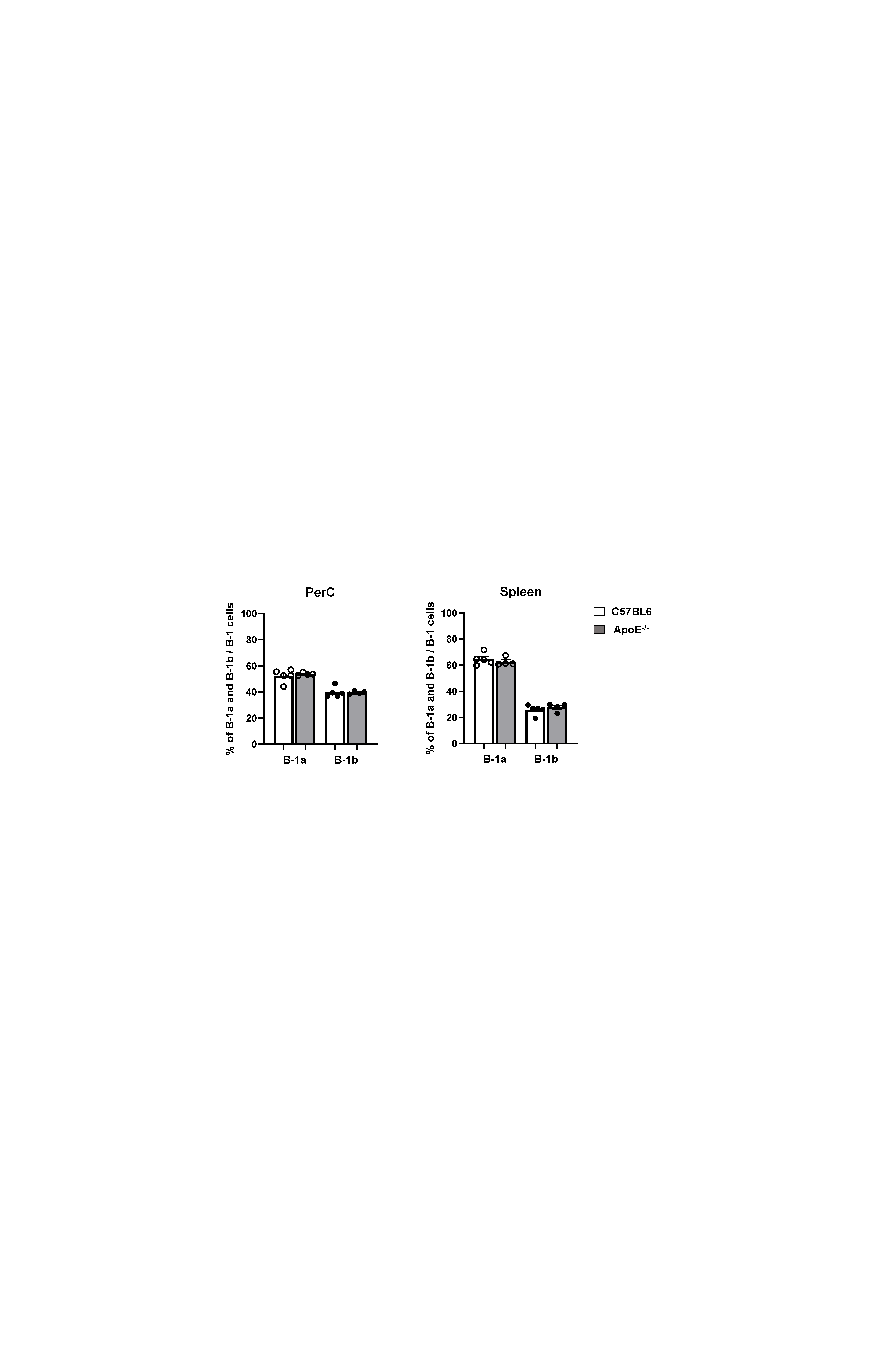

Supplement: Supplementary Figure 4 — Hyperlipidemia did not alter the frequencies of B-1a and B-1b cells at homeostasis. Flowcytometric analysis was performed in C57BL6 wild type and ApoE-/- mice at age of 10–12-week-old mice (n=5 mice/genotype) to understand the hyperlipidemic effect on B-1a and B-1b cells numbers in PerC and spleen. Results are represented in mean ± SEM, unpaired Mann-Whitney test was performed. [file Image_4.tif]
