# Supplementary material for: B-1b Cells Have Unique Functional Traits Compared to B-1a Cells at Homeostasis and in Aged Hyperlipidemic Mice With Atherosclerosis
Source: Front Immunol. 2022 Jul 22;13:909475. doi: 10.3389/fimmu.2022.909475 (PMC9353528; doi:10.3389/fimmu.2022.909475)
Supplement: Supplementary Table 2 — Replicate CDR-H3 amino acid sequences (those present more than once) and frequency present in sorted PerC B-1a, B-1b, splenic B-1a and B-1b. Asterisk (*) in PerC B-1a CDR-H3 sequence indicates stop codon. [file Table_2.pdf]

PerC B-1a (n=310 total sequences)			PerC B-1b (n=340 total sequences)			Spleen B-1a (n=214 total sequences)			Spleen B-1b (n=155 total sequences)		
**CDR-H3 Sequence**	**V-D-J**	**Number of CDR-H3 specific replicates (%)**	**CDR-H3 Sequence**	**V-D-J**	**Number of CDR-H3 specific replicates (%)**	**CDR-H3 Sequence**	**V-D-J**	**Number of CDR-H3 specific replicates (%)**	**CDR-H3 Sequence**	**V-D-J**	**Number of CDR-H3 specific replicates (%)**
AGDYDGYWYFDV	VH12 - DH2 - JH1	217 (70 %)	AGDRDGYWYFDV	VH12 - DH2 - JH1	91 (26.8 %)	AREVTTMYYFDY	VH1 - DH2 - JH2	30 (14 %)	AREDYYGSSYYFDY	VH1 - DH1 - JH2	15 (9.7 %)
ARSYYYGSSYYFDY	VH1 - DH1-1 - JH2	13 (4.2 %)	AREDYYGSSYYFDY	VH1 - DH2 - JH2	52 (15.3 %)	AGDYDGYWYFDV	VH12 - DH2 - JH1	20 (9.3 %)	TYYGNYENYTVDY	VH14 - DH2 - JH4	14 (9.0 %)
AGDRWGYWYFDV	VH12 - DH4 - JH1	13 (4.2 %)	AREVTTMYYFDY	VH1 - DH2 - JH2	18 (5.3 %)	MRYGDYWYFDV	VH11 - DH2 - JH1	8 (3.7 %)	ARAYGSSPYWYFDV	VH1 - DH1 - JH1	6 (3.9 %)
ARYYGNYWYFDV	VH1 - DH2 - JH1	6 (1.9 %)	AGDLLGYWYFDV	VH12 - DH2 - JH1	11 (3.2 %)	MRYSNYWYFDV	VH11 - DH2 - JH1	8 (3.7 %)	ARDDGYHGDWYFDV	VH1 - DH2 - JH1	4 (2.6 %)
AGDLLGYWYFDV	VH12 - DH2 - JH1	5 (1.6 %)	AREATTLDFFDY	VH1 - DH1 - JH2	5 (1.5 %)	AGDSDGYWYFDV	VH12 - DH2 - JH1	6 (2.8 %)	ATGSSFDY	VH1 - DH1 - JH2	3 (1.9 %)
ARDYYWYFDV	VH1 - DH2 - JH1	4 (1.3 %)	ARWDYDYPYRAMDY	VH1 - DH1- JH4	5 (1.5 %)	TRYYGSSAMDY	VH6 - DH1 - JH4	6 (2.8 %)	ASITTVVATPYYFDY	VH1 - DH1 - JH2	3 (1.9 %)
AGDSDGYWYFDV	VH12 - DH2 - JH1	4 (1.3 %)	ATGSSFDY	VH1 - DH1 - JH2	3 (0.9 %)	ASYYGNYWYFDV	VH1 - DH2 - JH1	4 (1.9 %)	MRYGDYWYFDV	VH11 - DH2 - JH1	3 (1.9 %)
AGDYYGYWYFDV	VH12 - DH1-1 - JH1	3 (1.0 %)	AGDTTGYWYFDV	VH12 - DH2 - JH1	3 (0.9 %)	ARSYYYGSSYYFDY	VH1 - DH1 - JH2	4 (1.9 %)	MRYSNYWYFDV	VH11 - DH2 - JH1	3 (1.9 %)
ASYYGNYWYFDV	VH1 - DH2 - JH1	2 (0.6 %)	*TYYGNYENYTVDY*	VH14 - DH1 - JH4	3 (0.9 %)	AGDYYGYWYFDV	VH12 - DH1 - JH1	4 (1.9 %)	AGDSDGYWYFDV	VH12 - DH2 - JH1	2 (1.3 %)
ATYYSNYWYFDV	VH1 - DH2 - JH1	2 (0.6 %)	ARRGNSYGNTFWYFDV	VH3 - DH1 - JH1	3 (0.9 %)	AKNDYGIYYYAMDY	VH2 - DH1 - JH4	4 (1.9 %)	AGDRNGYYHFDV	VH12 - DH2 - JH1	2 (1.3 %)
AREGDYYYGSSYWFAY	VH1 - DH1-1 - JH3	2 (0.6 %)	ARSYGYYAMDY	VH1 - DH2 - JH4	2 (0.6 %)	AKNEWLLERYAMDY	VH2 - DH2 - JH4	3 (1.4 %)	AGDRDGYWYFDV	VH12 - DH2 - JH1	2 (1.3 %)
MRYSNYWYFDV	VH11 - DH2 - JH1	2 (0.6 %)	ARGGFAY	VH1 - DH1 - JH3	2 (0.6 %)	ARSYGYYAMDY	VH1 - DH3 - JH4	2 (0.9 %)	TYYANSENYTVDY	VH14 - DH2 - JH4	2 (1.3 %)
AGDTTGYWYFDV	VH12 - DH4 - JH1	2 (0.6 %)	ASGLGVYWYFDV	VH1 - DH1 - JH1	2 (0.6 %)	ARYDYDEGDY	VH1 - DH2 - JH2	2 (0.9 %)	ARRGDYFDY	VH2 - DH1 - JH2	2 (1.3 %)
AGDRTGYWYFDV	VH12 - DH4 - JH1	2 (0.6 %)	ARSNYYAMDY	VH1 - DH2 - JH4	2 (0.6 %)	ARMGNYGSRYFDV	VH1 - DH1 - JH1	2 (0.9 %)			
AGDPYDGYYGFAY	VH12 - DH2 - JH3	2 (0.6 %)	AREDYYGSNYYFDY	VH1 - DH1 - JH2	2 (0.6 %)	ARGWLLRYYYAMDY	VH1 - DH2 - JH4	2 (0.9 %)			
AGDRDGYWYFDV	VH12 - DH2 - JH1	2 (0.6 %)	AGDNDGYYGFAY	VH12 - DH1 - JH3	2 (0.6 %)	ARRDGYGGFAY	VH1 - DH2 - JH3	2 (0.9 %)			
AKLR*XLL*L	VH2 - DH1-1 - JH2	2 (0.6 %)	AGDRDGYYSFDV	VH12 - DH1 - JH3	2 (0.6 %)	ANWAY	VH1 - DH4 - JH3	2 (0.9 %)			
ARYYYGSSYAMDY	VH7 - DH1-1 - JH4	2 (0.6 %)	AGDSDGYWYFDV	VH12 - DH2 - JH1	2 (0.6 %)	AREDYYGSSYYFDY	VH1 - DH1 - JH2	2 (0.9 %)			
			TTPSPNSNSFDY	VH14 - DH1 - JH2	2 (0.6 %)	VRDGITTVVAWDYYAMDY	VH10 - DH1 - JH4	2 (0.9 %)			
			TPHYYGSPWFAY	VH14 - DH2 - JH3	2 (0.6 %)	AGDPDGYYDFDV	VH12 - DH2 - JH1	2 (0.9 %)			
			TYYGNYESYVMDY	VH14 - DH1 - JH4	2 (0.6 %)	AGDKTGYWYFDV	VH12 - DH4 - JH1	2 (0.9 %)			
			ARNGGLWSYYAMDY	VH2 - DH1 - JH4	2 (0.6 %)	AGDRTGYWYFDV	VH12 - DH4 - JH1	2 (0.9 %)			
			TRYYGSSAMDY	VH6 - DH2 - JH4	2 (0.6 %)	ARRDYYYGSSYDYWYFDV	VH2 - DH1 - JH1	2 (0.9 %)			
			TYDYDWYFDV	VH6 - DH2 - JH1	2 (0.6 %)						
Frequency of replicate sequences:		**91.5 %**			**65.6 %**			**54.2 %**			**39.3 %**
